# Fungicidal Activity of AP10W, a Short Peptide Derived from AP-2 Complex Subunit mu-A, In Vitro and In Vivo

**DOI:** 10.3390/biom12070965

**Published:** 2022-07-10

**Authors:** Yi Gong, Haoyi Li, Fei Wu, Yishuai Li, Shicui Zhang

**Affiliations:** 1 Department of Marine Biology, Institute of Evolution & Marine Biodiversity, Ocean University of China, Qingdao 266003, China; gongyi0225@outlook.com (Y.G.); lihaoyi0601@163.com (H.L.); 18353686121@163.com (F.W.); 17569041837@163.com (Y.L.); 2 Laboratory for Marine Biology and Biotechnology, Pilot National Laboratory for Marine Science and Technology (Qingdao), Qingdao 266003, China

**Keywords:** antifungal peptide, wound healing, fungus, *Candida albicans*, *Aspergillus fumigatus*

## Abstract

With the increase in the incidence of fungal infections, and the restrictions of existing antifungal drugs, the development of novel antifungal agents is urgent. Here we prove that AP10W, a short peptide derived from AP-2 complex subunit mu-A, displays conspicuous antifungal activities against the main fungal pathogens of human infections *Candida albicans* and *Aspergillus fumigatus*. We also show that AP10W suppresses the fungal biofilm formation, and reduces the pre-established fungal biofilms. AP10W appears to exert its fungicidal activity through a mode of combined actions, including interaction with the fungal cell walls via laminarin, mannan and chitin, enhancement of cell wall permeabilization, induction of membrane depolarization, and increase in intracellular ROS generation. Importantly, we demonstrate that AP10W exhibits little toxicity towards mammalian fibroblasts, and effectively promotes the healing of wounded skins infected by *C. albicans*. These together indicate that AP10W is a new member of fungicidal agents. It also suggests that AP10W has a considerable potential for future development as a novel antifungal drug.

## 1. Introduction

Fungi are spore-producing eukaryotic organisms that live by decomposing and absorbing the organic material, in which they grow, comprising the mushrooms, molds, mildews, smuts, rusts, and yeasts. Fungal infections, or mycoses, have become a world-wide ever-growing threat to human health. For example, systemic fungal infections cause some 1.5 million deaths each year globally [1]. Fungal pathogens usually colonize human skin, mouth, intestines, and mucous membranes, and their uncontrolled growth and release of toxic products can cause a variety of infections in humans, ranging from superficial and subcutaneous life quality-debilitating infections affecting the skin, keratinous tissues and mucous membranes [2], to systemic infections of the brain, heart, lungs, liver, spleen, and kidneys that can be life-threatening [3]. These infections are of particular concern in the patients with autoimmune diseases, organ transplants and chemotherapy [4]. The main fungal pathogens of human infections include *Candida albicans*, and *Aspergillus fumigatus* [5]. *C. albicans* is the main agent responsible for mucosal diseases, and *A. fumigatus* for most allergic fungal diseases [5]. Although a few antibiotics such as amphotericin B, triazoles (fluconazole, itraconazole, and voriconazole), and echinocandins (micafungin and anidulafungin) are available to treat fungal infections [6], most of them, especially azoles and fluconazole, are found to be toxic to both animal and humans. Therefore, the availability of effective and safe antifungal therapies remains rather limited. Moreover, the emergence of antifungal resistance has recently been documented [7]. These all prompt an urgent need for the development of new antifungal agents with high efficiency and low toxicity.

Antimicrobial peptides (AMPs), as components of the first-line defense of host, are present in virtually all living beings [8,9,10,11], and can kill fungal cells and prevent biofilm formation [12,13,14,15]. AMPs with antifungal activity are often called antifungal peptides (AFPs). Due to the rapid microbicidal effects, broad-spectrum activities, extensive mechanisms of action, and low likelihood to induce resistance, AMPs have been regarded as the ideal candidates for the development of antimicrobial drugs, including AFPs [16,17,18]. 

Our previous study has shown that the peptide AP10W consisting of 10 residues, derived from AP-2 complex subunit mu-A, has antibacterial activity against several drug-sensitive and drug-resistant bacteria, and is not cytotoxic to human red blood cells and RAW264.7 cells [19]. We wonder if AP10W possesses antifungal activity. The present study is thus performed to test the antifungal activities and mechanisms of AP10W against *C. albicans* and *A. fumigatus*, the main fungal pathogens of human infections, and to evaluate its ability to promote *C. albicans*-infected wound healing in mice. 

## 2. Material and Methods

### 2.1. Fungal Strains and Culture

Stock cultures of *C. albicans* (SC5314) and *A. fumigatus* (Af293) maintained at −80 ℃ were used in our experiments. For recovery, *C. albicans* was cultured in yeast peptone dextrose (YPD) medium consisting of 1% Yeast Extract, 2% Peptone and 2% Glucose at 30 °C for 12 h, and *A. fumigatus* cultured in YPD agar consisting of 1% Yeast Extract, 2% Peptone, 2% Glucose and 2% agar at 30 °C for 48 h [20,21,22]. After recovery, *A. fumigatus* spores in agar surface were collected by inoculation loops and suspended in sterile water. The spore solution was vortexed, and filtered through five layers of sterile degreased cotton to obtain a homogenous spore suspension [18]. Both *C. albicans* and *A. fumigatus* were harvested by centrifugation at 6000× *g* at room temperature for 5 min. After washing three times with sterile water, they were resuspended in sterile water, adjusted to a density of 1 × 10^5^ cells/mL for *C. albicans* and 1 × 10^5^ spores/mL for *A. fumigatus*, and used for the following experiments. For convenience, the concentration of cells/mL was adopted for both *C. albicans* and *A. fumigatus* below.

### 2.2. Peptide Synthesis

Both AP10W and FITC-labeled AP10W, as well as biotin-labeled AP10W, were synthesized by Shanghai Sangon Biotech using the standard solid-phase 9-fluorenylmethoxy carbonyl (FMOC) method. Due to the common appearance of C-terminal amidation in AMPs [23], the C-termini of the peptides synthesized were thus amidated. All the peptides were purified >95% by high-performance liquid chromatography (HPLC) and verified by mass spectrometer (Icms-2010a). They were dissolved in sterile water, and stored at −80 °C until used. 

### 2.3. Fungal Killing Assay

The fungicidal activity of AP10W was examined by colony formation assay. Aliquots of 20 μL fungal suspension (1 × 10^5^ cells/mL) were mixed with 100 μL solution with different concentrations of AP10W, yielding a final concentration of 0 (control), 0.25, 0.5, 1, 2, 4, 8, 16 μg/mL, respectively, and incubated at 25 °C for 1 h. Each of the fungal mixtures was plated onto 3 YPD agar plates (20 μL/plate), and incubated at 30 °C for 24 to 48 h. The colonies in each plate were counted, and the percent of fungicidal activity was calculated by the formula: [number of colonies (control-test)/number of colonies (control)] × 100 (n = 3). Minimum fungicidal concentrations (MFC) were determined as the lowest peptide concentration that killed at least 99.9% of the fungal cells. 

### 2.4. Assay for Prevention of Fungal Biofilm Formation

The ability of AP10W to prevent biofilm formation was studied in 96-well plates using the crystal violet staining assay [21]. In brief, *C. albicans* and *A. fumigatus* were diluted with YPD medium to a density of 1 × 10^6^ cells/mL. In each well of a 96-well plate, aliquots of 10 μL fungal suspension (1 × 10^6^ cells/mL) were mixed with 50 μL solution with different concentrations of AP10W (ranging from 0 to 2 × MFC), and then in each well was added 140 μL YPD medium. After incubation at 30 °C for 24 h, the planktonic (free-floating) fungi were removed by washing with sterile water. The biofilms were photographed under an inverted fluorescence microscope (Leica DMI3000 B, Weztlar, Germany), and then dyed with 50 μL 0.1% crystal violet dye for 15 min. The dyed biofilms were washed with sterile water to remove the excess dye, and then 70% alcohol was added to dissolve the dye. The biomass of the biofilms was measured via the absorbance at 595 nm by a Multiskan MK3 microplate reader (Thermo Fisher Scientific, Waltham, MA, USA). Each test was performed in quintuplicate. 

### 2.5. Assay for Viability of Established Fungal Biofilms

The viability of established fungal biofilms following peptide AP10W treatment was assessed by the method of de Breij et al. [9]. Both *C. albicans* and *A. fumigatus* were diluted to 1 × 10^6^ cells/mL using YPD medium, and 100 μL of fungal suspension was added to each well of a 96-well plate. After incubation at 30 °C for 24 h to allow biofilm formation, each well was washed with sterile water. Subsequently, aliquots of 50 μL of AP10W solution with different concentrations ranging from 0 to 2 × MFC were added to corresponding wells. After incubation at room temperature for 2 h, the viability of the biofilm was measured as above, and an image of the biofilm was similarly taken. Each test was performed in quintuplicate.

### 2.6. Assay for Affinity of AP10W to Fungus and Permeability Change

The affinity of AP10W to fungal cells and cell wall permeability change were tested as described by Wang et al. [22]. The fungus *C. albicans* was selected as a representative of the experiment. The culture and collection of *C. albicans* were the same as described above. After washing 3 times with sterile water, the fungal pellets were suspended in sterile water, and adjusted to a density of 1 × 10^9^ cells/mL. FITC-labeled AP10W was then added to the fungal suspensions, yielding a final concentration of 4 μg/mL. Then, rTRX was labeled with FITC by the method of Wang et al. [24]. For negative control and blank control, the fungus was mixed with FITC-labeled rTRX (a final concentration of 4 μg/mL, which is equivalent to that of AP10W) or sterile water alone. The mixtures were incubated at room temperature for 2 h, and fixed with 20 μM PI solution under dark conditions at 4 °C for 15 min. The free FITC-labeled peptide and PI dye were removed by centrifugation, and the antifade mounting medium (Beyotime) was added to the samples to make a smear. The fungal cells stained by PI and the binding of FITC-labeled AP10W to the cells were both observed and photographed under an Olympus BX51 fluorescence microscope (Olympus, Tokyo, Japan) with a 488 and 535 nm band pass filter for FITC and PI excitation, respectively.

### 2.7. Assay for Binding of AP10W to Fungal Cell Wall Components

The binding of AP10W to the fungal cell wall components of laminarin (β-1,3-glucan polymer; Solarbio, Beijing, China), mannan (mannose polymer; Solarbio, Beijing, China) and chitin (Poly-(1→4)-β-N-acetyl-D-glucosamine; Sigma-Aldrich, Saint Louis, MO, USA) was detected by enzyme-linked immunosorbent assay (ELISA) as described by Wang et al. [24]. In brief, an aliquot of 50 μL laminarin, mannan or chitin (50 μg/mL) was applied to each well of a 96-well microplate, and air dried at 16 °C overnight. The wells were blocked with 200 μL of 10 mg/mL BSA in PBS (pH7.4) at 37 °C for 2 h. After rinsing once with PBST, a total of 50 μL PBS with 1 mg/mL BSA and different concentrations of biotin-labeled AP10W or BSA (ranging from 0 to 64 μg/mL) were added into each well, and incubated at 25 °C for 3 h. The wells were washed five times with PBST, and then incubated with 100 μL of streptavidin-HRP (CWBIO) that was diluted to 1:5000 with 1 mg/mL BSA in PBS at 25 °C for 1 h. Subsequently, the wells were washed five times with PBST, added with 75 μL of 0.4 mg/mL O-phenylenediamine (Amresco, Solon, OH, USA) in the buffer consisting of 51.4 mM Na_2_HPO_4_, 24.3 mM citric acid, and 0.045% H_2_O_2_ (pH5), and reacted at 37 °C for 10 min. Finally, 25 μL of 2 M H_2_SO_4_ was added into each well to terminate the reaction, and the absorbance at 492 nm was measured by a microplate reader.

### 2.8. Membrane Depolarization Assay

Membrane depolarization activity of AP10W was measured with the membrane potential-sensitive dye 3,3′-dipropylthiacarbocyanine iodide (DiSC_3_-5, Sigma-Aldrich) as described by Ni et al. [25]. The fungal cells of *C. albicans* and *A. fumigatus* were washed in 5 mM HEPES buffer (pH7.4) with 20 mM glucose, and resuspended in 5 mM HEPES buffer with 20 mM glucose and 100 mM KCl, giving a concentration of 1 × 10^7^ cells/mL. An aliquot of 100 μL of the fungal suspensions was applied to a well of a 96-well plate. A stock solution of DiSC_3_-5 was added to the fungal suspensions, giving a final concentration of 0.5 μM, and subjected to incubation at room temperature for 30 min to get a steady baseline of fluorescence intensity. The fungal suspensions were then mixed with 100 μL 20 μg/mL or 50 μg/mL of AP10W solution. A HEPES buffer containing 20 mM glucose was used as control. After incubation at room temperature for 10 min, the changes in fluorescence intensity were recorded continuously for 30 min with a GENios plus spectrofluorometer (Tecan, Männedorf, Switzerland) at an excitation wavelength of 622 nm and an emission wavelength of 670 nm.

### 2.9. Reactive Oxygen Species Assay

The fungi *C. albicans* and *A. fumigatus* were resuspended in the corresponding medium with 10 μM DCFH2-DA, yielding a density of 1 × 10^7^ cells/mL. After incubation at 37 °C for 30 min, both the fungi were collected by centrifugation at 6000× *g* at room temperature for 10 min. The fungal cells were washed three times with sterile water, resuspended in 1 mL of 20 μg/mL or 50 μg/mL of AP10W solution, respectively [26]. For positive control, the fungal cells were resuspended in 1 mL 50 μg/mL Rosup (Beyotime, Shanghai, China), a compound mixture that is able to considerably increase ROS levels in cells within 30 min. For blank control, the cells were resuspended in 1 mL PBS solution. The fungal suspensions were incubated at 25 °C for 1 h, and the fluorescence intensities recorded immediately with a spectrofluorimeter at an excitation wavelength of 488 nm and emission wavelength of 525 nm.

### 2.10. MTT Assay

The cytotoxicity of AP10W to the human embryonic lung fibroblast (HELF) cells (a gift of Dr. Bin Wang in the Medical School, Qingdao University) and the mouse embryonic fibroblast 3T3-L1 cells (a gift of Dr. Jingfeng Wang in the College of Food Science and Technology, Ocean University of China) were measured by using the 3-[4,5-dimethyl-2-thiazolyl]-2,5-diphenyl-2-H-tetrazolium bromide (MTT) dye method of Gong et al. [19]. Both HELF cells and 3T3-L1 cells were inoculated into 96-well plates (5 × 10^3^ cells/well) with a medium containing 10% fetal bovine serum, and incubated in a humidified incubator with 5% CO_2_ at 37 °C for 24 h. After removing the medium, the cells were incubated in 200 μL serum-free medium with different concentrations (0, 12.5, 25, 50 μg/mL) of AP10W for 4 h, and then 20 μL MTT solution (5 mg/mL) was added into each well, and incubated for another 4 h. Finally, the solution in each well was discarded, and 100 μL of pure dimethyl sulfoxide (DMSO; Solarbio) was added into each well to dissolve the methylzan crystals. The OD values were measured at 492 nm under a microplate reader. The percent cell viability versus the control was calculated as follows: Cell viability (%) = (OD_AP10W_ − OD_Blank_)/(OD_Control_ − OD_Blank_) × 100% (n =3), in which OD_AP10W_ is the OD value of the cells treated with AP10W, OD_Blank_ the OD value of blank control, and OD_Control_ the OD value of the cells treated without AP10W.

### 2.11. Creation of Mouse Wound Model and Wound Healing Assay

The animal experiments were approved by the Ethics Committee of the Laboratory Animal Administration of Shandong province (animal permit SCXK 20190003). The specific pathogen-free (SPF) male ICR mice (*Mus musculus*), aged 6 weeks (no. 370726220100017986), were purchased from Jinan Pengyue Laboratory Animal Breeding Co. Ltd., and housed one per cage in an environmentally controlled atmosphere (temperature 25 °C) with a 12-h light/dark cycle. They were given free access to water and diet, and provided with shredded wood flour bedding. The mice were given 7 days to adapt to the laboratory environment before the beginning of the experiments.

A clinically relevant model suitable for elucidating the pathophysiology underlying skin injury was created to investigate the antifungal activity of AP10W in vivo. The protocol was based on the method of Zhou et al. [27]. The mice were randomly divided into 3 groups before skin resection by staff not involved in the experiment (n = 9/group). The mice were anesthetized by intraperitoneal injection with 2% sodium pentobarbital. Following shaving off the dorsal hair using an electric clipper, a full thickness wound was created in the back skin of each mouse using a biopsy punch with 8-mm-diameter. Each wound in the mice of two groups was inoculated with 50 μL of *C. albicans* (1 × 10^9^ cells/mL), except for the blank control group. One hour later, 50 μL of 8 μg/mL AP10W was applied to the wound of the AP10W treatment group, and 50 μL of sterile saline was applied to the wound of the non-treatment group. The mice in the two groups were processed similarly once per day until day 5. The mice in the blank control group were subjected to no disposal. Wounds were photographed, and recorded using a camera at 0, 3, 5, 8, and 10 days after injury. The wound area was calculated, and analyzed with ImageJ software and presented as a percentage of the day 0 value.

### 2.12. Assessment of Colony Formation and Gram Staining

On day 3 after infection, two mice were randomly selected from each group, and humanely sacrificed by euthanasia using CO_2_ asphyxiation. The tissue of 1.2 cm × 1.2 cm wounded skin was dissected out from each mouse, and bisected. One part of the skin tissues was used for assessment of colony formation of wound tissues and the other used for Gram staining.

For assessment of colony formation, each group of the skin tissues was placed in 200 μL of double-distilled water, homogenized, and centrifugated at 6000× *g* at room temperature for 5 min [28]. The supernatants were collected, and adjusted with double-distilled water to 1 mL. Aliquots of 50 μL of each group suspension were plated onto Candida chromogenic medium plates (Hopebio, Qindao, China), and incubated at 30 °C for 48 h. For Gram staining, the skin tissue specimens were fixed in 4% paraformaldehyde (PFA), and stained with Gram stain [29], and photographed with an Olympus BX51 fluorescence microscope (Olympus, Tokyo, Japan). 

### 2.13. Statistical Analysis 

All the in vitro assays were performed at least in triplicate (technical replicates), and repeated 3 times (biologic replicates). Statistical differences were determined by one-way ANOVA and/or unpaired *t*-test using GraphPad Prism 5 (La Jolla, CA, USA). The value at *p* < 0.05 was considered significant. 

## 3. Results

### 3.1. AP10W Showed Antifungal Activity In Vitro 

**Figure 1:**
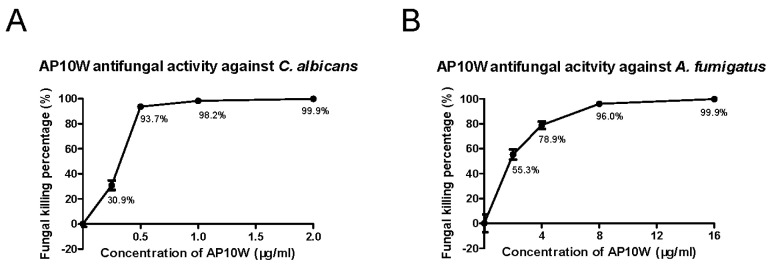
In vitro antifungal activity of AP10W. (**A**) Antifungal activity against *C. albicans* by AP10W (0, 0.25, 0.5, 1 and 2 μg/mL). (**B**) Antifungal activity against *A. fumigatus* by AP10W(0, 2, 4, 8 and 16 μg/mL). The sterile water was used as blank control. Data were expressed as means ± SD (n = 3) from three independent experiments.

The colony formation assay was used to determine the antifungal activity of AP10W. As shown in Figure 1, AP10W showed conspicuous antifungal activity against both the fungi tested *C. albicans* and *A. fumigatus*. The minimum fungicidal concentrations (MFCs) against *C. albicans* and *A. fumigatus* were 2 μg/mL and 16 μg/mL, respectively. 

### 3.2. AP10W Inhibited Fungal Biofilm Formation

**Figure 2:**
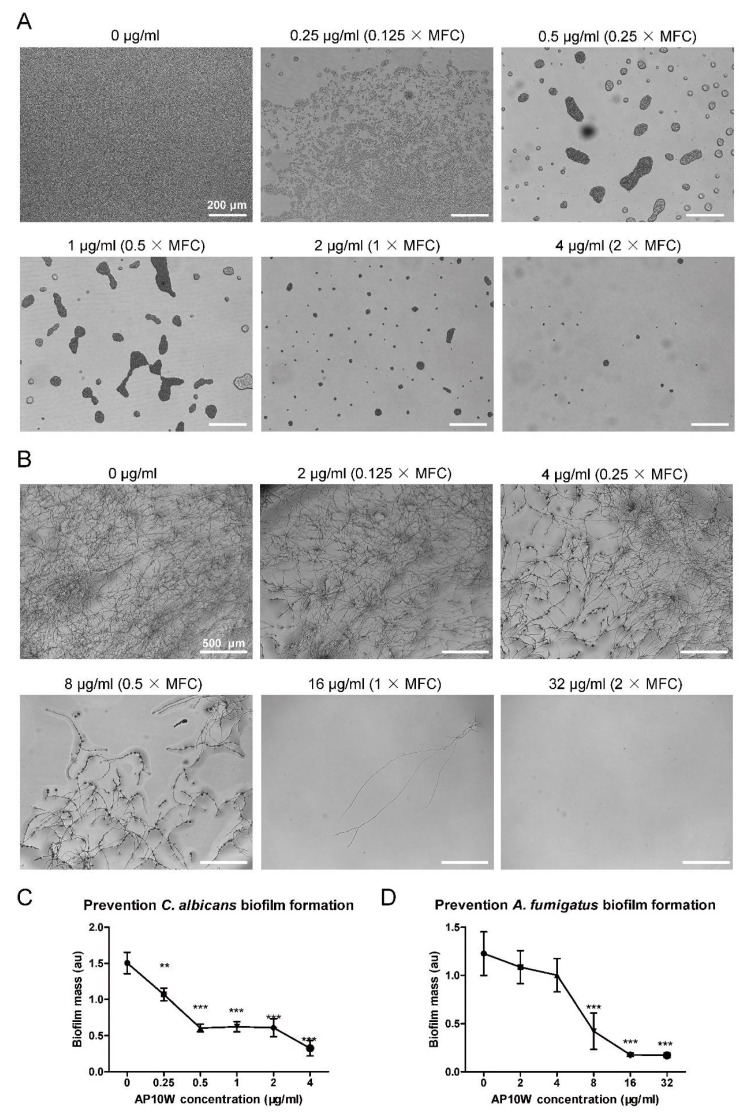
Growth inhibition of *C. albicans* and *A. fumigatus* biofilms by AP10W. (**A**) Microscope biofilm images of *C. albicans* incubated with different concentrations of AP10W (0 − 2 × MFC). Scale bars: 200 μm. (**B**) Microscope biofilm images of *A. fumigatus* incubated with different concentrations of AP10W (0 − 2 × MFC). Scale bars: 500 μm. (**C**) Inhibition of *C. albicans* biofilm formation by AP10W. (**D**) Inhibition of *A. fumigatus* biofilm formation by AP10W. Results are expressed as the biofilm mass, measured using crystal violet staining, in arbitrary units (au). Data are shown as means ± SD (n = 5) from three independent experiments. ** *p* < 0.01, *** *p* < 0.001.

As shown in Figure 2, AP10W inhibited the biofilm formation of both *C. albicans* and *A. fumigatus* in a dose-dependent manner. The biofilm mass was measured using crystal violet staining after images were taken. At the MFC and supra-MFC levels, AP10W almost completely suppressed the formation of *C. albicans* and *A. fumigatus* biofilms. We also observed that AP10W was able to prevent the initial attachment of *C. albicans* and *A. fumigatus* at concentration 0.125 × MFC (Figure 2A,B). Compared to control, AP10W significantly suppressed the biofilm formation of *C. albicans* and *A. fumigatus* at 0.25 μg/mL (0.125 × MFC) and 8 μg/mL (0.5 × MFC), respectively (Figure 2C,D).

### 3.3. AP10W Reduced Pre-Established Biofilms 

**Figure 3:**
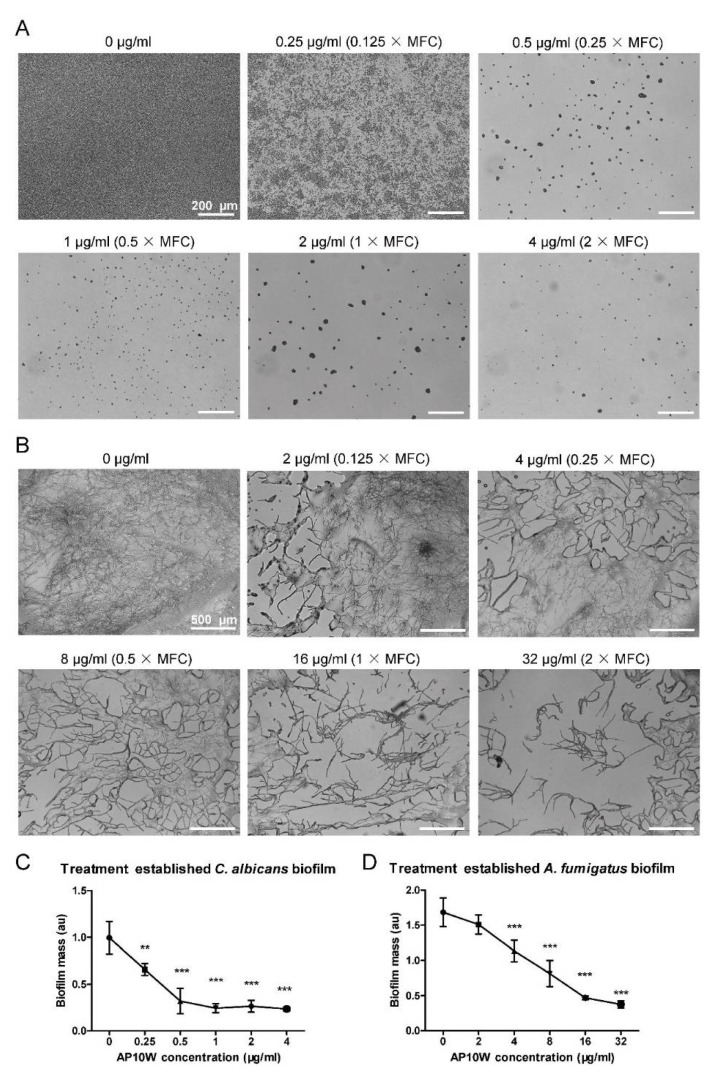
Destruction of *C. albicans* and *A. fumigatus* pre-established biofilms by AP10W. (**A**) Microscope images of *C. albicans* biofilm treated for two hours with different concentrations of AP10W (0 − 2 × MFC). Scale bars: 200 μm. (**B**) Microscope images of *A. fumigatus* biofilm treated for two hours with different concentrations of AP10W (0 − 2 × MFC). Scale bars: 500 μm. (**C**) AP10W reduces established *C. albicans* biofilm. (**D**) AP10W reduces established *A. fumigatus* biofilm. Results are expressed as the biofilm mass, measured using crystal violet staining, in arbitrary units (au). Data are shown as means ± SD (n = 5) from three independent experiments. ** *p* < 0.01, *** *p* < 0.001.

Fungal biofilms adhering to the medical materials is a serious clinical problem. Therefore, we evaluated the potential ability of AP10W to destroy the biofilms established by *C. albicans* and *A. fumigatus*. As shown in Figure 3, AP10W showed a dose-dependent destroying ability towards the biofilms pre-established by *C. albicans* and *A. fumigatus* within 2 h. At 0.125 × MFC concentration, AP10W reduced the adherence of both the fungi. AP10W almost completely eliminated the established biofilm of *C. albicans* at 0.5 × MFC, and removed a large proportion of the established biofilm of *A. fumigatus* at 2 × MFC (Figure 3A,B). Compared with control, AP10W significantly reduced the established biofilms of *C. albicans* and *A. fumigatus* at the concentrations of 0.25 μg/mL (0.125 × MFC) and 4 μg/mL (0.25 × MFC), respectively (Figure 3C,D).

### 3.4. AP10W Bound to C. albicans and Changed Cell Wall Permeability

**Figure 4:**
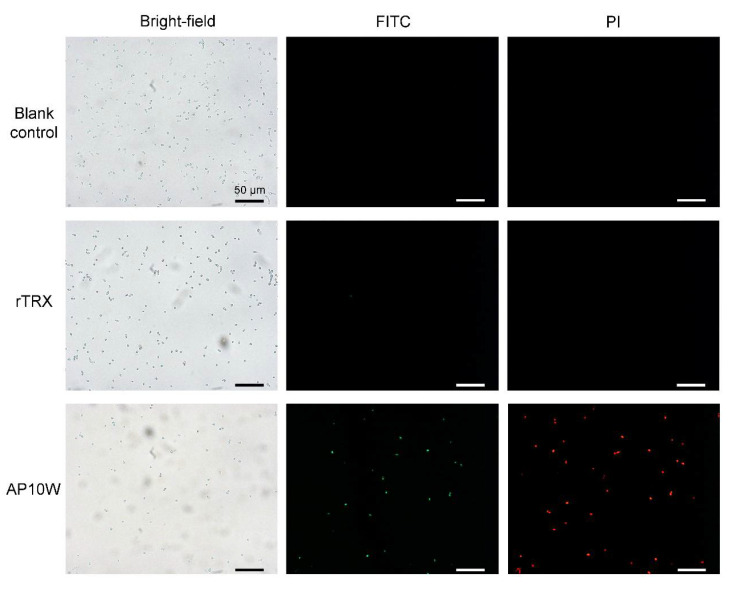
AP10W binds to *C. albicans* and causes cell wall permeabilization. rTRX was used as negative control, sterile water was used as blank control. Each test was performed in triplicate, and repeated three times. Scale bars: 50 μm.

As shown in Figure 4, *C. albicans* cells incubated with FITC-labeled AP10W showed green fluorescence, indicating the binding of AP10W to the fungal cells, whereas *C. albicans* cells incubated with FITC-rTRX showed little green fluorescence, indicating no interaction between rTRX and the fungal cells. In addition, AP10W caused changes in the cell wall permeability of *C. albicans* as PI, which only enters cells with the compromised cell walls, and induced intense red fluorescence in *C. albicans* cells. By contrast, rTRX induced no red fluorescence in *C. albicans* cells, and caused no cell wall permeabilization. These indicated that AP10W specifically interacted with *C. albicans* cells and induced cell wall permeabilization.

### 3.5. AP10W Bound to Laminarin, Mannan and Chitin

**Figure 5:**
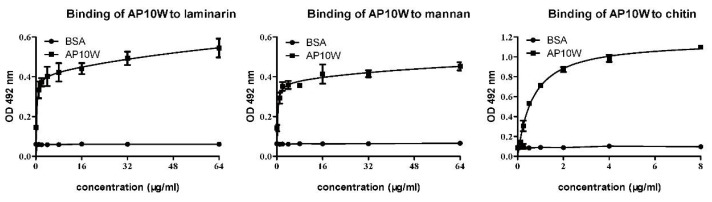
ELISA analysis of the affinity of AP10W to the laminarin, mannan and chitin. BSA served as control. Data are shown as means ± SD (n = 3). OD, optical density. The data are from three independent experiments performed in triplicate.

An ELISA was used to test if AP10W could bind to the fungal cell wall component’s laminarin, mannan and chitin. As shown in Figure 5, AP10W bound to both laminarin and mannan, as well as chitin, in a dose-dependent manner, whereas BSA used as negative control showed little binding to laminarin, mannan and chitin. These findings suggested that AP10W might interact with the fungi via their cell wall components laminarin, mannan and chitin.

### 3.6. AP10W Induced Fungal Membrane Depolarization

**Figure 6:**
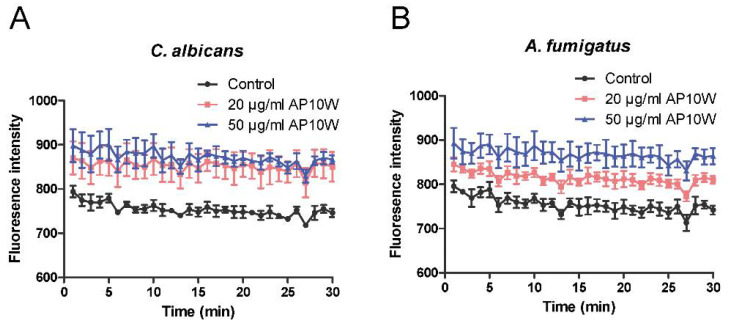
Fungal membrane depolarization. Depolarization of fungal cell membranes were detected using DiSC_3_-5 (excitation, 622 nm; emission, 670 nm). (**A**) The fluorescence intensity of *C. albicans* treated with different concentrations of AP10W. (**B**) The fluorescence intensity of *A. fumigatus* treated with different concentrations of AP10W. HEPES buffer containing 20 mM glucose was used as control. Data were expressed as means ± SD (n = 3) from three independent experiments.

The membrane depolarization activity of AP10W was assayed using DiSC_3_-5, a potential-dependent distributional fluorescent dye. As shown in Figure 6, the fluorescence intensities of both *C. albicans* and *A. fumigatus* cells treated with AP10W were increased, compared with control. These findings indicated that AP10W caused depolarization of the plasma membranes of *C. albicans* and *A. fumigatus*.

### 3.7. AP10W Induced ROS Production

**Figure 7:**
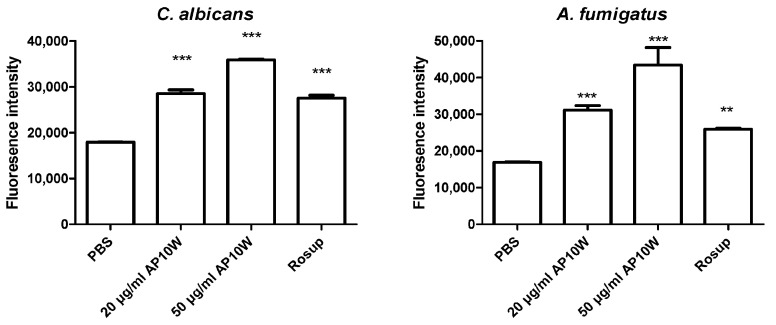
Effects of AP10W on intracellular ROS levels. The fungi *C. albicans* and *A. fumigatus* were treated with AP10W. Rosup, a compound mixture, can significantly increase ROS levels in cells within 30 min. PBS was used as control. Data were expressed as means ± SD (n = 3) from three independent experiments. ** *p* < 0.01, *** *p* < 0.001.

High intracellular ROS levels can cause cell apoptosis or necrosis. When *C. albicans* and *A. fumigatus* cells were treated with AP10W, their intracellular ROS levels were significantly increased, compared with control. In addition, high concentration of AP10W induced more intracellular ROS production (Figure 7). These findings suggested that AP10W induced apoptosis/necrosis of the fungal cells via increased production of intracellular ROS.

### 3.8. AP10W Was Not Toxic to Fibroblasts

We have previously shown that AP10W is toxic to murine RAW264.7 cells and human red blood cells. Here we tested the toxicity of AP10W to mammalian fibroblasts HELF cells and 3T3-L1 cells. As shown in Table 1, AP10W showed little cytotoxicity to either HELF cells or 3T3-L1 cells even at a concentration as high as 50 μg/mL. These findings indicated that AP10W was not toxic to mammalian fibroblasts, making it possible to treat wounded skin.

### 3.9. AP10W Promoted C. albicans-Infected Wound Healing

**Figure 8:**
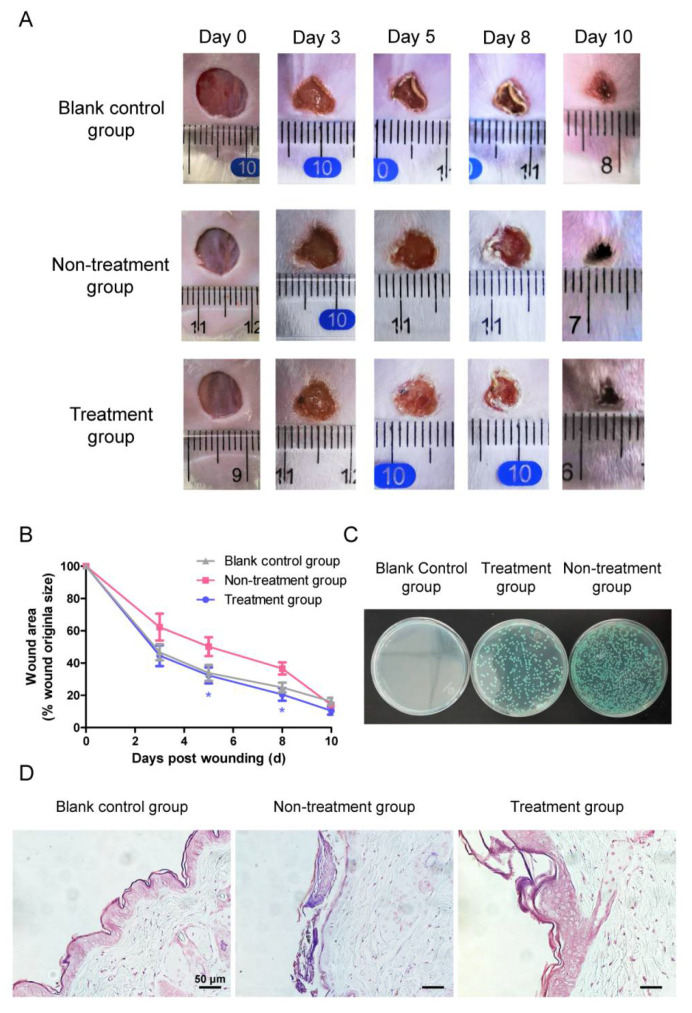
In vivo antifungal action of AP10W for *C. albicans*-infected wound in mice. (**A**) Representative photographs of wounds at 0, 3, 5, 8, and 10 days post wound. Blank control group: no *C. albicans*-infected, no treatment; Non-treatment group: *C. albicans*-infected; sterile saline treatment; Treatment group: *C. albicans*-infected; AP10W (8 μg/mL) treatment. Infection wounds with AP10W treatment showed accelerated healing compared to the infection group. (**B**) The area of the wounds was quantified by ImageJ software over multiple time points. Values represent means ± SEM (n = 7/group). Statistical differences between the treatment group and non-treatment group were assessed using unpaired t-test, * *p* < 0.05. (**C**) Different groups of wound skin tissue suspension were cultured in the chromogenic screening medium. *C. albicans* appeared as green smooth colonies. (**D**) Evaluation of wound by Gram staining of tissues. Wound skin tissue sections of three groups (blank control, non-treatment and treatment group) were Gram stained on day 3 (n = 2). Gram-positive *C. albicans* are indicated by purple. *C. albicans* were reduced in treatment group compared to the infection group. Scale bars: 50 μm.

Next, we tested the ability of AP10W to promote wound healing using mouse wound models. The photographs of the wounds were taken on days 0, 3, 5, 8, and 10, respectively, to measure the wound healing. It was found that application of AP10W to the mice wounds significantly enhanced the wound closure, compared to the non-treatment group (Figure 8A,B). The wound areas in the mice treated with AP10W were reduced to about 45%, 32%, 21%, and 10% of the original sizes at 3, 5, 8 and 10 days, respectively, which contrasted sharply to those of 62%, 50%, 37%, and 14% of the original sizes in non-treatment group (wounds were infected with *C. albicans*, but not treated with AP10W), and to those of 47%, 34%, 35% and 16% of the original sizes in the blank control group (wounds were neither infected with *C. albicans* nor treated with AP10W) at the same time points. The wounds in the mice of the blank control group and treatment group both started forming a scab on day 3, while the wounds in the mice of the non-treatment group remained inflamed and purulent on day 3, and the wound sizes were obviously larger than those of the blank control group and treatment group. These showed that AP10W promoted the closure and healing of skin wounds infected with *C. albicans*. 

A colony formation assay showed that both the tissue extracts of the mice from the treatment group and non-treatment group formed green smooth colonies, the typical characteristics of *C. albicans* cultured on screening medium (Figure 8C). By contrast, the tissue extracts of the mice from the blank control produced no colonies. In addition, the mice in the treatment group had remarkably lowered fungal loads, compared with those in non-treatment group. Gram staining of *C. albicans* is positive with a purple color. As shown in Figure 8D, no purple colored dots were seen in the skin tissues of the mice from the blank control group, indicating that *C. albicans* was not present. By contrast, purple colored dots were observed in the skin tissues of the mice from both the non-treatment group and treatment group, but the dots in the tissues of the treatment group mice were much less than those in the tissues of the non-treatment group mice, indicating that the *C. albicans* number in the tissues of treatment group mice was reduced, compared with the non-treatment group. This provided additional evidence that AP10W promoted the healing of skin wounds infected with *C. albicans*. 

## 4. Discussion

Despite the increase in the incidence of fungal infections, and the limitation of existing antifungal agents, only a few new antifungal drugs have been introduced in recent years. Moreover, the emergence of antifungal drug resistance strengthens the need for the development of novel antifungal molecules [30]. Furthermore, as fungal cells are eukaryotic, thus the development of selective antifungals is difficult [31], posing more challenges to modern medicine. In this study, we demonstrate that the AP2M1A-derived peptide AP10W inhibits the growth of the main fungal pathogens of human infections *C. albicans* and *A. fumigatus*. Pathogenic biofilms, often overlooked in medicinal research, are an important cause of chronic and recurrent infections [32]. We also demonstrate that AP10W suppresses the biofilm formation of both *C. albicans* and *A. fumigatus*, and destroys the biofilms pre-established by *C. albicans* and *A. fumigatus*. These together indicate that AP10W is a new member of fungicidal molecules capable of killing *C. albicans* and *A. fumigatus*, and reducing the biofilms formed by them.

The modes of action of antifungal drugs are generally associated with disruption of the fungal cell wall, damage of plasma membrane, disturbance of intracellular components, or induction of ROS production [33,34,35,36]. Here we show that AP10W interacts with the fungi via their cell wall components laminarin, mannan and chitin, which can lead to disruption or destabilization of the fungal cell wall. We also show that AP10W causes the fungal cell wall permeabilization, which can disrupt fungal cell walls. In addition, we show that AP10W induces both the fungal membrane depolarization and ROS generation, which are able to trigger apoptosis/necrosis of fungal cells. It is thus obvious that AP10W exerts its fungicidal activity through a mode of combined actions.

Given that AP10W shows fungicidal activity in vitro, we thus evaluate its antifungal activity in vivo using mouse wound models. We show first that AP10W is non-toxic to the fibroblasts HELF cells and 3T3-L1 cells, and thus it can be used safely. We then show that AP10W promotes the closure and healing of skin wounds infected with *C. albicans*. Additionally, we also show that AP10W significantly reduces the loads of *C. albicans* on the wounded skins. These indicate that AP10W functions as a fungicidal agent, even in a complex physiological condition, making it a lead peptide for the future development of novel antifungal drugs.

In conclusion, our study highlights AP10W as a new antifungal peptide capable of killing *C. albicans* and *A. fumigatus*, reducing the biofilms formed by them, and promoting the healing of wounded skins infected by *C. albicans*. It also shows that AP10W has considerable potential for future development as a novel antifungal drug.

## Figures and Tables

**Table 1:** The percent viability of 3T3-L1 cells and HELF cells incubated with different concentrations of AP10W. Data were expressed as means ± SD (n = 3) from three independent experiments.

AP10W Concentration (ug/mL)	Percent Viability of 3T3-L1	Percent Viability of HELF
0	100	100
12.5	126 ± 3	115 ± 6
25	115 ± 8	108 ± 1
50	111 ± 13	104 ± 4

## Data Availability

The data presented in this study are available on request from the corresponding author.
